# Small molecule inhibitors as adjuvants in cancer immunotherapy: enhancing efficacy and overcoming resistance

**DOI:** 10.3389/fimmu.2024.1444452

**Published:** 2024-08-05

**Authors:** Xiaolin Wu, Nuan Feng, Chao Wang, Hongfei Jiang, Zhu Guo

**Affiliations:** ^1^ The Affiliated Hospital of Qingdao University, Qingdao University, Qingdao, China; ^2^ Department of Orthopedics, The Affiliated Hospital of Qingdao University, Qingdao, China; ^3^ Department of Nutrition, Peking University People’s Hospital, Qingdao, China; ^4^ Women and Children’s Hospital, Qingdao University, Qingdao, China; ^5^ Department of Spinal Surgery, Affiliated Hospital of Qingdao University, Qingdao, China

**Keywords:** adjuvant, adjuvant therapy, neoadjuvant therapy, anti-tumor drug, cancer immunotherapy, small molecule inhibitor

## Abstract

Adjuvant therapy is essential in cancer treatment to enhance primary treatment effectiveness, reduce adverse effects, and prevent recurrence. Small molecule inhibitors as adjuvants in cancer immunotherapy aim to harness their immunomodulatory properties to optimize treatment outcomes. By modulating the tumor microenvironment, enhancing immune cell function, and increasing tumor sensitivity to immunotherapy, small molecule inhibitors have the potential to improve patient responses. This review discusses the evolving use of small molecule inhibitors as adjuvants in cancer treatment, highlighting their role in enhancing the efficacy of immunotherapy and the opportunities for advancing cancer therapies in the future.

## 1 Introduction

Adjuvant therapy in cancer treatment refers to the use of additional treatments such as chemotherapy, radiation, or targeted therapies following primary treatments like surgery. The main objectives of adjuvant therapy are to enhance the effectiveness of the primary treatment, reduce adverse effects, and prevent disease recurrence (1–3). This approach targets residual cancer cells post-surgery, helping to reduce the risk of cancer returning and spreading, and thereby improving the overall success rates of cancer eradication (4, 5). In the context of cancer immunotherapy, small molecule inhibitors serve as immune adjuvants. These inhibitors aim to modulate the tumor microenvironment, enhance immune cell function, and increase tumor sensitivity to immunotherapy (6, 7). By leveraging their immunomodulatory properties, small molecule inhibitors can optimize treatment outcomes, improve patient responses, and provide new opportunities for advancing cancer therapies (8).

In cancer immunotherapy, the concept of using small molecule inhibitors as adjuvants involves leveraging the immunomodulatory effects of these drugs to enhance the effectiveness of immunotherapy. For example, small molecule inhibitors can modulate the tumor microenvironment, boost immune cell function, increase tumor sensitivity to immunotherapy, and achieve better treatment outcomes (9–11). Using small molecule inhibitors as adjuvants in cancer treatment is a rapidly evolving and expanding field. By researching how small molecule inhibitors interact with immunotherapy, optimizing treatment regimens, predicting patient responses to treatment, it can provide more opportunities and improvements for future cancer treatments. In this comprehensive review, we delve into the evolving role of small molecule inhibitors as adjuvants in cancer immunotherapy, exploring their mechanisms of action, clinical applications, and potential for improving treatment outcomes.

## 2 Mechanisms of action of small molecule inhibitors in cancer immunotherapy

Small molecule inhibitors play a significant role in cancer immunotherapy by targeting specific pathways and molecules involved in regulating the immune response to tumors. These inhibitors act through various mechanisms to modulate the tumor microenvironment and enhance the anti-tumor immune response. Some common mechanisms of action of small molecule inhibitors in cancer immunotherapy have been summarized.

### 2.1 Immune checkpoint blockade

Immune checkpoint blockade is a cutting-edge cancer immunotherapy that targets molecules like CTLA-4 and PD-1 to activate T cells and boost anti-tumor immunity. By blocking inhibitory signals, checkpoint inhibitors unleash the immune system to recognize and eliminate cancer cells (12, 13). Monoclonal antibodies targeting CTLA-4, such as ipilimumab, disrupt this inhibitory signal, enhancing T cell activation and anti-tumor immune responses (14). Small molecule inhibitors, on the other hand, are designed to interfere with intracellular signaling pathways, thus modulating immune responses indirectly. PD-1, expressed on T cells upon activation, interacts with PD-L1 to inhibit T cell function (15). Small molecule inhibitors targeting the PD-1/PD-L1 pathway can modulate intracellular signaling pathways, leading to T cell activation and immune-mediated tumor cell killing (16). By targeting these key immune checkpoint molecules with small molecule inhibitors, we can modulate immune responses to overcome tumor-induced immune suppression and expand the therapeutic landscape in cancer immunotherapy.

### 2.2 Signal transduction pathways

Signal transduction pathways play a critical role in regulating immune responses in cancer, including immune cell activation, proliferation, and effector functions. Small molecule inhibitors targeting key signaling molecules within these pathways have emerged as promising adjuvants in cancer immunotherapy (17) (18). For instance, inhibitors of the PI3K-Akt-mTOR pathway can modulate T cell activation and differentiation, enhancing anti-tumor immunity (19). Inhibitors of the MAPK pathway, such as MEK inhibitors, can regulate T cell function and cytokine production to optimize anti-tumor immune responses (20). Additionally, inhibitors of the NF-κB pathway can modulate inflammatory responses and immune cell activation. By selectively targeting specific nodes within these signaling pathways, small molecule inhibitors can fine-tune immune responses to promote effective anti-tumor immunity (21). Understanding the intricate interplay of signal transduction pathways and harnessing the therapeutic potential of small molecule inhibitors offer exciting avenues to expand the therapeutic landscape of cancer immunotherapy and improve patient outcomes.

### 2.3 Enhancing immune cell infiltration

Enhancing immune cell infiltration into tumors is a critical mechanism by which small molecule inhibitors act as adjuvants in cancer immunotherapy. Effective infiltration of immune cells into the tumor microenvironment is essential for mounting a robust anti-tumor immune response. Small molecule inhibitors target key pathways and mechanisms, such as angiogenesis, extracellular matrix remodeling, and immune cell infiltration, to enhance the immune response within the tumor microenvironment.

#### 2.3.1 Targeting angiogenic pathways

Tumor growth and progression are heavily dependent on the formation of new blood vessels, a process known as angiogenesis. Tumors secrete vascular endothelial growth factor (VEGF) to promote angiogenesis, which also creates an abnormal and disorganized vascular network that impedes immune cell infiltration. Small molecule inhibitors, such as those targeting VEGF receptors (VEGFR), can normalize the tumor vasculature. By inhibiting VEGF signaling, these inhibitors can reduce the formation of new blood vessels, disrupt existing abnormal vessels, and improve the overall vascular structure within the tumor. This normalization of the tumor vasculature facilitates better penetration and infiltration of immune cells, such as cytotoxic T lymphocytes (CTLs) and natural killer (NK) cells, into the tumor microenvironment (22).

#### 2.3.2 Modulating the extracellular matrix

The extracellular matrix (ECM) within tumors often presents a physical barrier to immune cell infiltration. Small molecule inhibitors can modulate components of the ECM to enhance immune cell penetration. For instance, inhibitors targeting enzymes such as matrix metalloproteinases (MMPs) can degrade ECM components, thereby reducing the physical barriers that prevent immune cells from reaching the tumor core. By altering the ECM composition, these inhibitors create pathways for immune cells to infiltrate more effectively (23).

#### 2.3.3 Reducing immunosuppressive cells

The tumor microenvironment often contains a high number of immunosuppressive cells, such as regulatory T cells (Tregs) and myeloid-derived suppressor cells (MDSCs), which inhibit the activity and infiltration of effector immune cells. Small molecule inhibitors can selectively target and reduce the population of these immunosuppressive cells. For example, inhibitors of the colony-stimulating factor-1 receptor (CSF-1R) can decrease the number of MDSCs, thereby reducing their suppressive effects on immune cell infiltration and function. This reduction in immunosuppressive cells enhances the ability of effector immune cells to infiltrate the tumor and exert their anti-tumor effects (24).

#### 2.3.4 Enhancing chemokine signaling

Chemokines are signaling molecules that guide the migration of immune cells to sites of inflammation, including tumors. Small molecule inhibitors can enhance chemokine signaling pathways to promote the recruitment and infiltration of immune cells into tumors. For instance, inhibitors that upregulate the expression of chemokines such as CXCL9 and CXCL10 can attract more CTLs to the tumor site. By increasing the concentration of these chemokines in the tumor microenvironment, small molecule inhibitors enhance the directional migration of immune cells into the tumor, improving their infiltration and subsequent anti-tumor activity (25).

### 2.4 Immunomodulation

Small molecule inhibitors, by targeting immunomodulatory pathways, can shape the immune landscape within the tumor microenvironment to bolster immune-mediated tumor eradication (26). These inhibitors can modulate the activity of various immune cell populations, such as T cells, regulatory T cells, and myeloid-derived suppressor cells, to tip the balance in favor of anti-tumor immune responses. Furthermore, small molecule inhibitors can impact cytokine signaling networks, influencing the immune cell functions and interactions critical for mounting effective anti-tumor immune responses (27). By fine-tuning immune responses through targeted immunomodulation, small molecule inhibitors can overcome immune evasion mechanisms employed by tumors and enhance the efficacy of cancer immunotherapy. Leveraging the power of immunomodulation in conjunction with other therapeutic strategies, such as immune checkpoint blockade or targeted therapies, offers a multifaceted approach to expand the therapeutic landscape of cancer immunotherapy and improve patient outcomes.

By targeting these key pathways and mechanisms, small molecule inhibitors can synergize with immunotherapy approaches to improve treatment outcomes in cancer patients. Further research into the precise mechanisms of action of small molecule inhibitors in cancer immunotherapy holds promise for developing more effective and targeted cancer treatments.

## 3 Clinical applications of small molecule inhibitors as adjuvants in cancer immunotherapy

Small molecule inhibitors have demonstrated promising clinical applications in cancer immunotherapy across various types of cancer (9). These inhibitors play a crucial role as adjuvants by enhancing immune responses, overcoming resistance mechanisms, and improving overall treatment outcomes. Their ability to modulate the tumor microenvironment and improve immune cell infiltration makes them valuable assets in combination with existing immunotherapy approaches.

For instance, studies have shown that small molecule inhibitors targeting VEGFR can normalize tumor vasculature, facilitating better immune cell infiltration and enhancing the effectiveness of immune checkpoint inhibitors in clinical settings. In a phase II clinical trial, the combination of the VEGFR inhibitor axitinib with the PD-1 inhibitor pembrolizumab showed significant improvement in response rates and overall survival in patients with metastatic renal cell carcinoma (28). Similarly, the PI3K inhibitor idelalisib has been used successfully in combination with rituximab for the treatment of relapsed chronic lymphocytic leukemia, demonstrating the potential of small molecule inhibitors in enhancing the efficacy of immunotherapy (29).

### 3.1 Combination therapy

Combining small molecule inhibitors with other immunotherapeutic approaches, such as immune checkpoint inhibitors or adoptive T cell therapy, offers a synergistic approach to amplify immune responses and overcome resistance mechanisms (30). By targeting distinct signaling pathways or immune checkpoints simultaneously, combination therapy has the potential to broaden the spectrum of anti-tumor immune responses and improve treatment outcomes. Furthermore, combining small molecule inhibitors with traditional cancer treatments like chemotherapy or radiation therapy can create a multifaceted attack on tumor cells, leading to more comprehensive and durable responses (31). The rational design of combination regimens that leverage the strengths of different therapeutic modalities holds promise in expanding the therapeutic landscape of cancer immunotherapy and addressing the challenges of immune evasion and tumor heterogeneity.

### 3.2 Overcoming resistance

Resistance mechanisms, such as immune evasion and tumor heterogeneity, can limit the success of immunotherapeutic approaches. Small molecule inhibitors can help overcome resistance by targeting pathways involved in immune evasion and tumor immune escape (32). By disrupting these critical signaling pathways in the tumor microenvironment, small molecule inhibitors can enhance immune cell infiltration, reprogram immune responses, and restore immune recognition of tumor cells (33). Additionally, combination therapies that incorporate small molecule inhibitors alongside immunotherapies or other treatments present a comprehensive strategy to combat resistance and enhance treatment outcomes. Overall, by targeting resistance mechanisms, small molecule inhibitors play a vital role in expanding the therapeutic landscape of cancer immunotherapy and improving patient responses to treatment.

### 3.3 Personalized medicine and adjuvant therapy

By leveraging small molecule inhibitors in cancer immunotherapy, personalized medicine aims to identify specific molecular targets or pathways unique to each patient’s tumor (34). This precision medicine approach allows for the selection of the most effective small molecule inhibitors based on the molecular characteristics of the tumor, genetic profile of the patient, and immune response (35). By customizing treatment regimens to match the individual tumor biology and immune landscape, personalized medicine maximizes therapeutic efficacy while minimizing side effects.

Additionally, overcoming drug resistance is a critical aspect of personalized medicine. Certain genetic changes caused by drug resistance can be precisely regulated through targeted small molecule inhibitors. By identifying and targeting these specific genetic alterations, small molecule inhibitors can help to overcome resistance and restore sensitivity to treatment. The use of small molecule inhibitors as adjuvants in cancer immunotherapy represents a promising strategy to expand the therapeutic landscape, overcome treatment resistance, and improve patient responses to immunotherapy.

### 3.4 New targets and indications

Small molecule inhibitors offer the potential to target novel pathways and molecular targets that have not been previously exploited in immunotherapeutic approaches. By identifying and leveraging these new targets, researchers can broaden the scope of immunotherapy strategies, address the challenges of treatment resistance, and enhance therapeutic efficacy (36). Additionally, the discovery of new indications for small molecule inhibitors in cancer immunotherapy opens up opportunities to treat a wider range of cancer types and patient populations (37). The pursuit of novel targets and indications for small molecule inhibitors in cancer immunotherapy holds great promise for expanding the therapeutic landscape and improving outcomes for individuals with cancer.

The clinical applications of small molecule inhibitors in cancer immunotherapy represent a rapidly evolving field, with ongoing research focusing on optimizing treatment regimens, identifying biomarkers, and expanding the therapeutic potential of these inhibitors in various cancer types (38). As our understanding of the tumor microenvironment and immune response continues to advance, small molecule inhibitors are poised to play a pivotal role in shaping the future of cancer immunotherapy.

## 4 Examples of small molecule inhibitors with immunomodulatory effects in cancer immunotherapy

Small molecule inhibitors are increasingly recognized for their role in cancer immunotherapy due to their ability to modulate immune responses and enhance anti-tumor activity. These inhibitors target specific proteins and pathways involved in cancer cell proliferation and survival, as well as the tumor microenvironment, which can suppress the immune system (39). By inhibiting these targets, small molecule inhibitors can restore or enhance the immune system’s ability to recognize and destroy cancer cells.

Some well-known examples include Vemurafenib, which targets BRAF and modulates the tumor microenvironment to promote T cell infiltration in melanoma (40); Dasatinib, which targets multiple tyrosine kinases and enhances immune cell function in leukemia and solid tumors (41); and Ibrutinib, which inhibits BTK and modulates B-cell receptor signaling in B-cell malignancies (42). Other notable inhibitors, such as Sunitinib and Pazopanib, target multiple receptor tyrosine kinases and have shown efficacy in reducing regulatory T cells and myeloid-derived suppressor cells, thereby enhancing the overall immune response in various cancers (43, 44). These small molecule inhibitors, by targeting critical pathways involved in immune regulation and tumor growth, offer significant potential to improve the effectiveness of cancer immunotherapies and patient outcomes. In **Table 1**, selected examples of these small molecule inhibitors, which have been proven to exert excellent immunomodulatory effects, have been summarized with discussion of their action mechanisms.

**Table 1:** Selected examples of small molecule inhibitors with immunomodulatory effects and their action mechanisms.

Small Molecule Inhibitor	Target	Mechanism of Action	Cancer Types	Refs
Vemurafenib (Zelboraf)	BRAF	Inhibits mutated BRAF protein; modulates tumor microenvironment and promotes T cell infiltration	Melanoma	(40)
Dasatinib (Sprycel)	BCR-ABL, SRC family kinases	Inhibits multiple tyrosine kinases; modulates immune cell function and enhances anti-tumor immune response	Leukemia, solid tumors	(41)
Imatinib (Gleevec)	BCR-ABL	Inhibits BCR-ABL tyrosine kinase; alters tumor microenvironment and influences immune response	Chronic myeloid leukemia (CML), GIST	(42)
Sunitinib (Sutent)	VEGFR, PDGFR, KIT	Multi-targeted receptor tyrosine kinase inhibitor; reduces Tregs and MDSCs, enhancing immune response	Renal cell carcinoma, gastrointestinal stromal tumors	(43)
Idelalisib (Zydelig)	PI3Kδ	Inhibits PI3Kδ; affects immune cell subsets and tumor microenvironment	B-cell malignancies (e.g., CLL, FL)	(44)
Ibrutinib (Imbruvica)	BTK	Inhibits BTK; modulates B-cell receptor signaling and immune cell function	B-cell malignancies (e.g., CLL, MCL)	(45)
Acalabrutinib (Calquence)	BTK	Inhibits BTK; similar to ibrutinib but with potentially fewer off-target effects	B-cell malignancies (e.g., CLL, MCL)	(46)
Cabozantinib (Cometriq)	VEGFR, MET, AXL	Inhibits multiple tyrosine kinases; reduces immunosuppressive cells and enhances anti-tumor immunity	Renal cell carcinoma, medullary thyroid cancer	(47)
Pazopanib (Votrient)	VEGFR, PDGFR, KIT	Inhibits multiple receptor tyrosine kinases; modulates immune cell infiltration and function	Renal cell carcinoma, soft tissue sarcoma	(48)
Sorafenib (Nexavar)	RAF, VEGFR, PDGFR	Multi-kinase inhibitor; affects tumor angiogenesis and immune cell function	Hepatocellular carcinoma, renal cell carcinoma	(49)
Crizotinib (Xalkori)	ALK, ROS1	Inhibits ALK and ROS1; modulates tumor microenvironment and immune response	Non-small cell lung cancer (NSCLC)	(50)
Ceritinib (Zykadia)	ALK	Inhibits ALK; similar to crizotinib but more potent	Non-small cell lung cancer (NSCLC)	(51)
Alectinib (Alecensa)	ALK	Inhibits ALK; effective in crizotinib-resistant cases	Non-small cell lung cancer (NSCLC)	(52)
Brigatinib (Alunbrig)	ALK, EGFR	Inhibits ALK and EGFR; modulates immune cell function and tumor microenvironment	Non-small cell lung cancer (NSCLC)	(53)
Nilotinib (Tasigna)	BCR-ABL	Inhibits BCR-ABL tyrosine kinase; affects immune responses and tumor microenvironment	Chronic myeloid leukemia (CML)	(54)
Erlotinib (Tarceva)	EGFR	Inhibits EGFR; modulates tumor cell growth and immune cell infiltration	Non-small cell lung cancer (NSCLC), pancreatic cancer	(55)
Gefitinib (Iressa)	EGFR	Inhibits EGFR; affects tumor cell proliferation and immune responses	Non-small cell lung cancer (NSCLC)	(56)
Afatinib (Gilotrif)	EGFR, HER2	Inhibits EGFR and HER2; modulates immune cell function and tumor microenvironment	Non-small cell lung cancer (NSCLC)	(57)
Osimertinib (Tagrisso)	EGFR	Inhibits EGFR T790M mutation; modulates immune responses	Non-small cell lung cancer (NSCLC)	(58)
Venetoclax (Venclexta)	BCL-2	Inhibits BCL-2; promotes apoptosis of cancer cells and influences immune responses	Chronic lymphocytic leukemia (CLL), AML	(59)
Selinexor (Xpovio)	XPO1	Inhibits nuclear export protein XPO1; modulates immune responses and tumor cell growth	Multiple myeloma, diffuse large B-cell lymphoma	(60)
Alpelisib (Piqray)	PI3Kα	Inhibits PI3Kα; affects tumor cell proliferation and immune cell function	Breast cancer	(61)
Trametinib (Mekinist)	MEK	Inhibits MEK; affects tumor cell signaling and immune cell infiltration	Melanoma	(62)
Cobimetinib (Cotellic)	MEK	Inhibits MEK; similar to trametinib, enhances immune cell function	Melanoma	(63)
Dabrafenib (Tafinlar)	BRAF	Inhibits BRAF; similar to vemurafenib, affects immune cell function and tumor microenvironment	Melanoma	(64)
Everolimus (Afinitor)	mTOR	Inhibits mTOR; modulates immune responses and tumor cell proliferation	Renal cell carcinoma, breast cancer	(65)
Temsirolimus (Torisel)	mTOR	Inhibits mTOR; similar to everolimus, affects immune cell function	Renal cell carcinoma	(66)
Ruxolitinib (Jakafi)	JAK1, JAK2	Inhibits JAK1/2; modulates immune cell function and cytokine signaling	Myelofibrosis, polycythemia vera	(67)
Tofacitinib (Xeljanz)	JAK1, JAK3	Inhibits JAK1/3; affects immune cell signaling and function	Rheumatoid arthritis, being investigated for cancer	(68)
Abemaciclib (Verzenio)	CDK4, CDK6	Inhibits CDK4/6; affects cell cycle progression and modulates immune responses	Breast cancer	(69)

These examples highlight the diverse mechanisms of action and clinical applications of small molecule inhibitors with immunomodulatory effects in cancer immunotherapy. By targeting specific pathways involved in immune regulation and tumor growth, these inhibitors have the potential to enhance the efficacy of immunotherapy and improve outcomes for cancer patients.

## 5 Challenges and future directions in small molecule inhibitors as adjuvants in cancer immunotherapy

### 5.1 Resistance mechanisms

One of the challenges in using small molecule inhibitors as adjuvants in cancer immunotherapy is the development of resistance mechanisms by tumors (32). Tumors can acquire mutations or activate alternative signaling pathways to bypass the effects of these inhibitors. For example, resistance to BTK inhibitors like ibrutinib in B-cell malignancies often involves mutations in the BTK binding site or activation of PLCγ2 signaling (70). Future research should focus on understanding these resistance mechanisms and developing strategies to overcome them, such as combination therapies with other targeted agents or immunotherapies.

### 5.2 Specificity and off-target effects

Small molecule inhibitors may have off-target effects on normal cells, leading to toxicities and adverse effects (71). For instance, the multi-kinase inhibitor sunitinib has been associated with cardiotoxicity and hypertension due to its off-target effects on other kinases (72). Improving the specificity of these inhibitors to target tumor cells while sparing healthy tissues is crucial for minimizing toxicity and improving the safety profile of combination therapies.

### 5.3 Biomarker identification

Biomarkers that predict response to small molecule inhibitors as adjuvants in cancer immunotherapy are still evolving (73). Identifying reliable biomarkers to predict response to small molecule inhibitors as adjuvants in cancer immunotherapy is another unique challenge. For example, PD-L1 expression is a known biomarker for response to checkpoint inhibitors, but similar biomarkers for small molecule inhibitors are still being explored (74). Research efforts should prioritize the discovery and validation of biomarkers that can guide treatment selection and monitor response to therapy.

### 5.4 Optimal dosing and scheduling

Determining the optimal dosing and scheduling of small molecule inhibitors in combination with immunotherapy is essential for maximizing therapeutic efficacy while minimizing toxicity. This is particularly important for inhibitors that may have cumulative toxicities when used in combination regimens. Studies have shown that staggered dosing schedules can reduce toxicity and improve outcomes in combination therapies involving kinase inhibitors and immunotherapies (75).

### 5.5 Combination therapy strategies

Developing rational combination therapy strategies with small molecule inhibitors and immunotherapy agents is a complex and evolving field (30). For instance, combining VEGFR inhibitors with checkpoint inhibitors has shown promise in preclinical models, but optimal combinations and sequences need to be established through clinical trials (76). Future directions should explore novel combinations, target multiple pathways simultaneously, and leverage advances in tumor immunology to enhance the anti-tumor immune response and overcome treatment resistance.

### 5.6 Translational research and clinical trials

Translating preclinical findings into clinical practice and conducting well-designed clinical trials are essential for evaluating the safety and efficacy of small molecule inhibitors as adjuvants in cancer immunotherapy (77). Future research directions should prioritize rigorous clinical testing and validation of promising combination therapies.

Overall, addressing these challenges and advancing research efforts in biomarker identification, treatment optimization, combination therapy strategies, and clinical trial design will be critical for harnessing the full potential of small molecule inhibitors as adjuvants in cancer immunotherapy and improving outcomes for cancer patients. Collaborative efforts between researchers, clinicians, and industry stakeholders will be essential for driving progress in this rapidly evolving field.

## 6 Conclusion

In conclusion, small molecule inhibitors have emerged as promising adjuvants in cancer immunotherapy, offering the potential to enhance the anti-tumor immune response and improve treatment outcomes for cancer patients. By targeting specific signaling pathways involved in tumor growth and immune evasion, these inhibitors can modulate the tumor microenvironment, sensitize tumors to immune-mediated destruction, and potentiate the effects of immunotherapy agents.

Despite the significant progress in the development and clinical use of small molecule inhibitors in cancer treatment, several challenges remain to be addressed. Resistance mechanisms, off-target effects, biomarker identification, optimal dosing and scheduling, as well as rational combination therapy strategies are important considerations that need to be carefully addressed in future research and clinical practice. Moving forward, future directions in small molecule inhibitors as adjuvants in cancer immunotherapy should focus on overcoming resistance mechanisms, improving specificity and safety profiles, identifying predictive biomarkers, optimizing treatment regimens, developing innovative combination therapies, and conducting robust translational research and clinical trials.

It is essential that collaborative efforts and multidisciplinary approaches be employed to advance the field of small molecule inhibitors in cancer immunotherapy. By addressing these challenges and pursuing innovative research strategies, we can harness the full potential of small molecule inhibitors to improve patient outcomes, enhance treatment response rates, and ultimately pave the way for more effective and personalized cancer therapies in the future.

## Author contributions

HJ: Writing – original draft, Writing – review & editing. XW: Writing – original draft, Writing – review & editing. NF: Data curation, Writing – review & editing. CW: Data curation, Writing – review & editing. ZG: Writing – original draft, Writing – review & editing.
